# Relating Cognitive-Activating Instruction and Metacognitive Self-Regulation to Mathematics Performance and Self-Efficacy: A Process-Modelling Study

**DOI:** 10.3390/bs16061029

**Published:** 2026-06-19

**Authors:** Ioannis G. Katsantonis

**Affiliations:** Department of Psychology, Panteion University of Social and Political Sciences, 176 71 Athens, Greece; ikatsanton@edu.panteion.gr

**Keywords:** metacognition, metacognitive self-regulation, mathematics self-efficacy, cognitive activation, mathematics performance, PISA 2022

## Abstract

This study examined the processes linking cognitively activating mathematics instruction to self-efficacy via metacognitive self-regulation. A sequential mediation model was tested whereby cognitive-activating instruction operationalised as mathematical argumentation was specified as being associated with metacognitive self-regulation, which, in turn, was estimated to be associated with mathematics performance and mathematics self-efficacy. Data from 6403 adolescents (49.76% females) from Greece’s PISA 2022 dataset were utilised. Latent variables were constructed from the student questionnaire items to capture cognitive activation, metacognitive self-regulation, and self-efficacy. Structural equation modelling showed that cognitive activation was positively associated with metacognitive self-regulation, which, in turn, was positively associated with mathematics self-efficacy. Sequential mediation analysis indicated that cognitive-activating instruction was also directly linked to mathematics self-efficacy and indirectly through mathematics performance, supporting the role of performance as a source of mastery experiences. In brief, the findings imply that engaging students in cognitively activating activities is associated with better metacognitive self-regulation skills and higher mathematics self-efficacy, partly through mathematics performance, which is consistent with the mastery-experiences account.

## 1. Introduction

Mathematical competence, that is, the capability to take appropriate actions in response to mathematical challenges (Niss & Højgaard, 2019), is considered essential for both academic performance and successfully navigating everyday mathematical problems (OECD, 2023). Grounded in mathematical knowledge and skills, it provides students with a critical foundation to pursue science, technology, engineering, and mathematics (STEM) careers, which are key to future jobs in a fast-changing world shaped by technology. Beyond domain-specific skills, mathematics competence also cultivates soft skills (Wolf & McCoy, 2019). These are essential for future personal, educational, and economic success and becoming reflective, constructive, and active citizens in the 21st century (European Commission, 2022; OECD, 2023).

Despite the importance of mathematical competence, international evidence suggests a decline in mathematics performance among adolescents, which underscores the need for further studies that examine the processes associated with higher mathematics performance among adolescents (Granello et al., 2025). Importantly, this decline is situated within the broader developmental context of adolescence. Adolescence represents a developmental stage that involves not only notable physiological changes (Sawyer et al., 2018), but also changes in motivational and metacognitive processes that are associated with learning and performance (dos Santos Kawata et al., 2021; I. Katsantonis, 2024; I. Katsantonis & McLellan, 2023; Veenman et al., 2006). Within this developmental context, one key factor that has been consistently associated with mathematics performance is mathematics self-efficacy (Street et al., 2024). Self-efficacy has been shown to predict not only mathematics performance but also learning engagement (Tang et al., 2021). Additionally, mathematics self-efficacy holds the potential to close gender- and ethnicity-based gaps in mathematics performance (Yang et al., 2024). These findings suggest that, although the role of self-efficacy in promoting mathematics performance is well established, less attention has been paid to the instructional and cognitive factors associated with such self-efficacy beliefs in classroom contexts.

Despite the fact that mathematics self-efficacy can predict mathematics performance (Street et al., 2024), mathematics performance provides students with direct evidence of their competence through mastery experiences (Usher & Pajares, 2009). Hence, mathematics performance should be viewed not only as a final product but also as part of a process that requires moving beyond motivational processes such as self-efficacy and considering the role of self-regulatory skills. One set of skills linked with mathematics performance is metacognitive self-regulation (Lingel et al., 2019; Muncer et al., 2022; Xie et al., 2026).

While self-efficacy helps explain why students engage with mathematics tasks, understanding factors associated with performance in classroom contexts requires attention to metacognitive self-regulatory processes and the instructional practices that may be linked with them. Crucially, metacognitive self-regulation is shaped not only by maturational processes but also by classroom instructional practices (I. G. Katsantonis, 2025; Xie et al., 2026). One teaching strategy that supports self-regulation skills is cognitive activation, which involves instructional practices that challenge students’ thinking and make students recall prior knowledge, construct arguments, and link learning material to real-life practical examples (Baumert et al., 2010; Teig et al., 2019). Hence, it is important to examine the potential association of cognitive activation with metacognitive control and monitoring.

Despite the growing evidence linking cognitive activation, metacognitive self-regulation, mathematics performance and self-efficacy, these associations have been examined mostly through bivariate associations. Consequently, there is limited evidence on how these processes are structurally related within a single theoretically grounded framework, particularly with large-scale representative samples. Thus, the current study aims to contribute to the literature by testing a social-cognitive model in which cognitive-activating instruction is linked to mathematics self-efficacy via metacognitive self-regulation and mathematics performance using representative Greek data from the Programme for International Student Assessment.

### 1.1. Theoretical Framework

#### 1.1.1. Cognitive Activation as an Instructional Antecedent of Metacognition

Cognitive activation is a critical instructional strategy that encompasses a range of techniques such as linking new mathematics concepts with previously acquired knowledge (I. G. Katsantonis, 2025; Li et al., 2021), involving students in cognitively challenging tasks and engaging students in constructive discourse (Zuo et al., 2024). There is ample evidence that cognitive activation can be positively associated with mathematics performance (Förtsch et al., 2016; S. Liu & Yin, 2024; Zuo et al., 2024). Therefore, it is important to consider how cognitive activation can be linked with other critical antecedents of mathematics performance, such as metacognitive self-regulation.

The empirical literature regarding the associations between cognitive-activating instruction and metacognitive self-regulation is particularly sparse in large-scale, adolescent samples and process-oriented models. In a systematic review that synthesised classroom observation studies, it was reported that classroom environments that enabled students to think, plan, and evaluate had the capacity to activate metacognitive processes (Dignath & Veenman, 2021). Another study with older adolescents (aged 17 years old) reported a positive indirect pathway from cognitive activation to metacognitive regulation via self-efficacy and achievement emotions (boredom, enjoyment) (Ekatushabe et al., 2021). Overall, it can be understood that the evidence regarding the association between cognitive-activating instruction and metacognitive self-regulation is limited and it remains to be seen whether and how these factors are interrelated and can contribute collectively to mathematics performance and self-efficacy. In the next section, the link between metacognitive regulation and performance is discussed.

#### 1.1.2. Metacognitive Self-Regulation in Relation to Mathematics Performance

One theoretically relevant set of processes associated with mathematics performance is metacognition. Metacognition in the learning sciences is generally conceptualised as individuals’ capacity to monitor and regulate their cognitive activity whilst solving academic tasks (Efklides, 2008; I. Katsantonis, 2020; I. Katsantonis & McLellan, 2023; Metcalfe & Shimamura, 1996). Recent work emphasises that metacognition is a multicomponent construct that encompasses knowledge, feelings, and control of cognitive processing, which is called metacognitive skills or self-regulation (Efklides, 2006; Rivers, 2021). Metacognition also involves monitoring, that is, individuals’ capability to be aware of one’s knowledge about the task and to estimate the accuracy of one’s cognitive processing (Efklides, 2006). In particular, metacognitive self-regulation is a central component of self-regulated learning and academic adaptation (Panadero, 2017; Schunk, 2005). Metacognitive self-regulation usually includes the planning, monitoring, and evaluation of one’s progress during learning (Efklides, 2006; Pintrich et al., 1991; Schraw & Dennison, 1994). Metacognitive skills can be conceptualised either as domain-general (i.e., broader task-independent behavioural strategies) or domain-specific (i.e., focusing on a specific task) (Zhao et al., 2019). Since metacognition becomes more domain-general when children transition to adolescence (ages 10 to 13) (Geurten et al., 2018), the current study conceptualises metacognitive self-regulation as a domain-general skill.

Empirical work generally supports the importance of metacognition for mathematics performance, despite the picture being more nuanced. For instance, the meta-analytic study by Muncer et al. (2022) reported that the relationship between metacognitive processes and mathematics performance was moderately positive in adolescence, which indicates that metacognitive processes are indeed meaningful for performance outcomes. Research studies have revealed that metacognitive self-regulation skills were a good predictor of mathematics performance (Nelson & Fyfe, 2019; Wang et al., 2021). In this sense, students who can accurately regulate the quality of their understanding and work tend to produce stronger mathematics performance, which, in turn, may serve as the basis for stronger mathematics self-efficacy.

In sum, metacognitive regulation strategies have been associated with academic performance, which may be interpreted as relevant to mastery experiences and mathematics self-efficacy (Usher, 2009). Thus, in the next section, I discuss mathematics performance as an outcome of mathematics mastery experiences.

#### 1.1.3. Mathematics Self-Efficacy as an Outcome of Mastery Experiences

Grounded in social-cognitive theory (Bandura, 1997), mathematics self-efficacy refers to one’s belief in one’s capability to perform well in mathematics tasks (Ferla et al., 2009). A substantial body of research has conceptually and empirically positioned mathematics self-efficacy as a predictor of mathematics performance (Street et al., 2024; Yang et al., 2024). Within this dominant perspective, self-efficacy is treated by researchers as an antecedent of performance, which influences students’ effort, persistence, and learning behaviours (Madrilejos, 2025; Tang et al., 2021). However, Bandura’s (1997) social-cognitive theory posits that self-efficacy beliefs are constructed through the interpretation of prior experiences, with mastery experiences—i.e., previous performance—representing the most influential source [see also (Usher, 2009; Usher & Pajares, 2009)]. From this standpoint, mathematics performance should not be considered merely an outcome but a key process through which students form and recalibrate their beliefs about their mathematical capabilities.

For example, one longitudinal study showed that higher mathematics performance was positively associated with higher mathematics self-efficacy beliefs, which is consistent with the social-cognitive idea that successful performance provides competence-relevant feedback (R. Liu et al., 2024). Similarly, other longitudinal findings indicate that mathematics performance and mathematics self-efficacy are modestly positively linked, sometimes in a mutually reinforcing manner (Du et al., 2021). At the same time, there is longitudinal evidence showing that mathematics performance was not associated with mathematics self-efficacy (Schöber et al., 2018). Taken together, these findings suggest that although mathematics self-efficacy and performance are interlinked constructs, the potential predictive relationship from mathematics performance to mathematics self-efficacy requires further study.

In light of this lack of consensus, a cautious interpretation is warranted. Using cross-sectional PISA 2022 data, the current modelling of mathematics performance as a factor associated with self-efficacy reflects a theoretically grounded specification rather than a causal claim. Given the contradictory evidence in the literature, the findings should be interpreted as indicative of associations only, contributing to an ongoing debate about the relationship between performance and self-efficacy.

#### 1.1.4. The Present Study: An Integrative Framework for Mathematics Self-Efficacy

**Figure 1:**
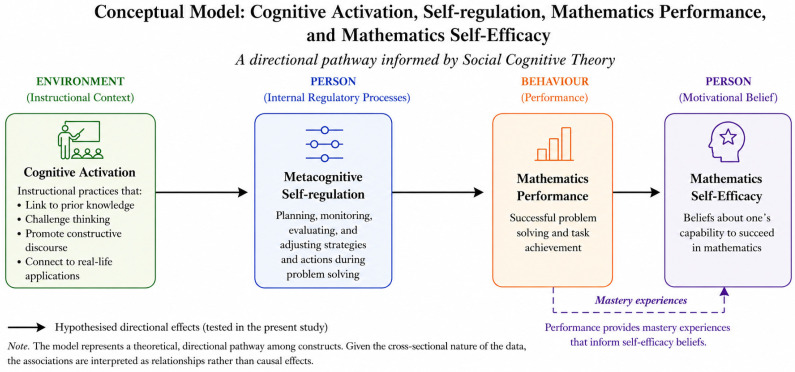
Social-cognitive conceptual model of the study.

Building on the above theoretical and empirical considerations, the current study proposes an integrative framework. The conceptual model is presented in Figure 1. The novelty of the present study lies in testing the full theoretically specified social-cognitive sequence from cognitive-activating instruction to metacognitive self-regulation, mathematics performance, and self-efficacy using nationally representative PISA 2022 data from Greece. In greater detail, the present study contributes to the literature in three important ways. First, the study advances prior research by modelling these constructs within a single sequential mediation framework rather than examining isolated bivariate associations. Second, it provides nationally representative evidence from Greek adolescents using the most recent PISA cycle focused on mathematics. Third, by specifying mathematics performance as part of the pathway to self-efficacy, the study offers a theoretically grounded test of a sequence consistent with social-cognitive theory and the mastery-experiences account of self-efficacy.

The overarching research question that the current study aims to address is the following:

**RQ1.**  *How are cognitive-activating instruction, metacognitive self-regulation, mathematics performance and self-efficacy linked together?*

Several hypotheses were derived from this research question. It is expected that cognitive-activating instruction in mathematics will be associated with metacognitive self-regulation (H1). Next, metacognitive self-regulation is hypothesised to associate with mathematics performance (H2), which in turn will be associated with mathematics self-efficacy (H3).

## 2. Materials and Methods

### 2.1. Data and Sample

Data from 6403 Greek adolescent students were drawn from the 2022 version of the Programme for International Student Assessment (PISA) that were collected by the Organization for Economic Co-Operation and Development (OECD) (OECD, 2023). The data collection commenced in Greece on 15 March and ended on 15 April 2022. The sample was composed of 49.76% female adolescents. Most participants (91.54%) spoke Greek as the main language at home and studied in Grade 10 (98.17%), that is, the first grade of Lyceum. The data are accessible from OECD at https://www.oecd.org/en/data/datasets/pisa-2022-database.html (accessed on 23 July 2024).

### 2.2. Measures

All item wordings are presented in Tables S1–S3 in the Supplemental Materials, along with the measurement model’s factor loadings.

#### 2.2.1. Mathematics Self-Efficacy

Seven items drawn from the student questionnaire module were utilised to measure adolescents’ mathematics self-efficacy. The prompt was “How confident do you feel about having to do the following mathematics tasks?”. Sample items include “Solving an equation like 2(x + 3) = (x + 3) (x − 3)” and “Finding the actual distance between two places on a map with a 1:10,000 scale”. The items were scored using a four-point Likert-type scale ranging from 1 “not at all confident” to 4 “very confident”. Cronbach’s alpha was 0.87 for this scale.

#### 2.2.2. Cognitive Activation: Mathematics Argumentation

The cognitive activation scale, labelled mathematics argumentation, reflected teaching practices of explaining, justifying, defending, and generating solutions. This is a modified version of the PISA 2022 cognitive activation: fostering reasoning measure (OECD, 2024b) because the original measure displayed limited validity for the Greek context based on the strength of the factor loadings and the fit indices from confirmatory factor analyses. The revised scale consisted of six items with the following question prompt “This school year, how often did your teacher do the following things in your mathematics lessons?”. Sample items include “The teacher asked us to explain how we solved a mathematics problem” and “The teacher encouraged us to think about how to solve mathematics problems in different ways than demonstrated in class”. The scoring scale ranged from 1 “never or almost never” to 5 “every lesson or almost every lesson”. Cronbach’s alpha for the revised mathematics argumentation scale was 0.85, indicating very good reliability.

#### 2.2.3. Metacognitive Regulation

Although PISA 2022 does not provide a direct measure of metacognitive regulation, it was possible to construct a brief proxy scale of global metacognitive self-regulation by identifying items that clearly map onto the control component of metacognition (Brown, 1980; Efklides, 2008). These items were conceptualised to reflect metacognitive self-regulation because they capture deliberate planning before action and monitoring/self-evaluation of performance outcomes. Consistent with metacognitive self-regulation frameworks (Schraw & Dennison, 1994; Sperling et al., 2002), behaviours such as pausing to consider the next course of action, evaluating completed work, and attending potential errors represent monitoring, checking, and self-evaluative aspects of cognitive control during task completion. Accordingly, aspects of metacognitive self-regulation were approximated using four items from the student questionnaire. The overall question prompt was “to what extent do you agree or disagree with the following statements?”. Sample items from the metacognitive self-regulation scale include “I like to make sure there are no mistakes” and “I carefully check homework before turning it in”. The four items were scored using a five-point Likert-type scale ranging from 1 “strongly disagree” to 5 “strongly agree”. Cronbach’s alpha index of reliability was 0.72, indicating good reliability. The measure should be interpreted as capturing broad metacognitive self-regulation tendencies, rather than a comprehensive assessment of metacognitive self-regulation.

#### 2.2.4. Mathematics Performance

Adolescents’ mathematics performance was measured using the PISA 2022 standardised assessment outcomes (OECD, 2023). The PISA 2022 adaptive cognitive assessment included a standardised assessment of adolescent students’ mathematics skills and specifically, tested their ability to reason mathematically and to formulate and apply mathematical concepts, strategies and reasoning in real-world contexts and domains (OECD, 2023). Mathematics scores were extracted as plausible values from Item Response Theory modelling (OECD, 2023). The PISA mathematics scores have no theoretical minimum or maximum but the average of the scale is 500 and 100 is the standard deviation across all participating countries (OECD, 2024a). The reliability of the mathematics performance scale was 0.89 for the Greek sample (OECD, 2024c).

### 2.3. Statistical Analyses

To evaluate common method bias, Harman’s single factor approach (Harman, 1967) and the bifactor modelling (Rodriguez et al., 2016) were utilised. That is, the percent of variance explained by the first unrotated factor in a minimum residual exploratory factor analysis was examined to evaluate whether common method bias was likely to be problematic. Additionally, the explained common variance coefficient (ECV) in bifactor modelling was computed to estimate the percent of variance explained by a general ‘method’ factor that was uncorrelated with the specific latent factors (i.e., metacognitive self-regulation, mathematics self-efficacy, argumentation and conceptual reasoning). Furthermore, the omega hierarchical (ω_H_) reliability coefficient was computed to evaluate whether a general ‘method’ factor might be reliable (Rodriguez et al., 2016). Because PISA mathematics performance was assessed via a standardised test rather than student self-report, the above procedures are interpreted as diagnostic checks for a general response pattern. The bifactor indices (i.e., ECV and ω_H_) were calculated using the lavaan package (Rosseel, 2012) and the BifactorIndicesCalculator package (Dueber, 2021) in R (R Core Team, 2023). Reliability of the scales was computed using the semTools package (Jorgensen et al., 2026).

Next, confirmatory factor analysis (CFA) and structural equation modelling (SEM) were implemented in Mplus (Muthén & Muthén, 2017). To account for the complex sampling design of the PISA survey, the command TYPE = COMPLEX was specified to adjust the estimates for the sampling weight and the clustering of the adolescent students within schools. The models were estimated using the robust maximum likelihood estimator (MLR), which addresses non-normality and is suitable for data with four or five response categories (Bandalos, 2014; Rhemtulla et al., 2012). The fit of the hypothesised latent variable models was evaluated using the conventional guidelines for the fit indices. Specifically, CFI and TLI values close to 0.95 accompanied by RMSEA and SRMR values below 0.06 were taken to indicate a good fitting model (Hu & Bentler, 1999).

## 3. Results

### 3.1. Descriptive Statistics and Correlations

Descriptive statistics were computed as mean scores for each variable and are presented in Table 1. As can be seen in Table 1, adolescents reported moderate levels of metacognitive self-regulation and perceived cognitive-activating instruction, and below-average mathematics self-efficacy. Metacognitive self-regulation was slightly above average, whereas the average mathematics performance in this sample was below the OECD average of 500 (OECD, 2023). All variables displayed skewness values near zero, indicating approximately normally distributed data.

**Figure 2:**
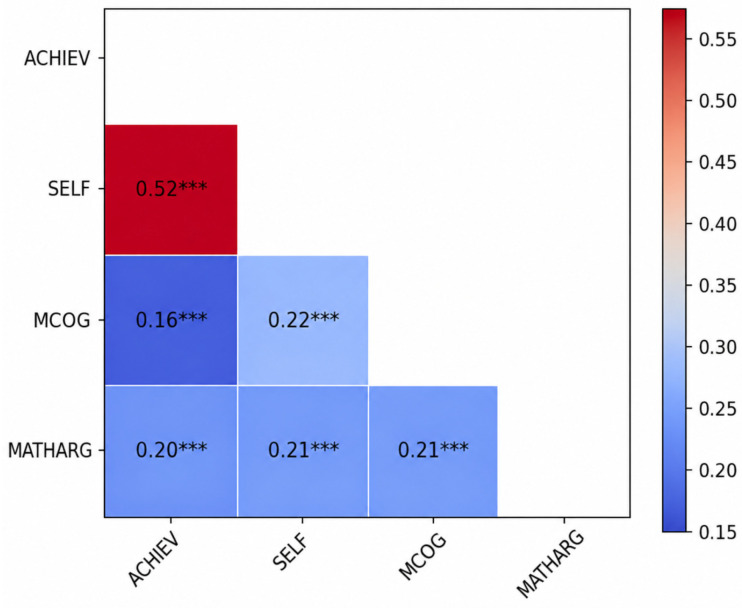
Heatmap showing the correlations between the study’s measures. Correlations were derived from the weighted and cluster-adjusted CFA measurement model. *** *p* < 0.001.

Based on the latent correlation analyses presented in Figure 2, several statistically significant associations were observed among the study variables. Most notably, mathematics self-efficacy was strongly correlated with mathematics performance (ACHIEV; *r* = 0.52, *p* < 0.001). In addition, mathematics self-efficacy demonstrated modest but consistent associations with metacognitive self-regulation (MCOG; *r* = 0.22, *p* < 0.001) and cognitive activation in mathematics instruction (MATHARG; *r* = 0.21, *p* < 0.001). Mathematics performance was also positively related to both metacognitive self-regulation (*r* = 0.16, *p* < 0.001) and cognitive activation (*r* = 0.20, *p* < 0.001). Finally, a moderate association was observed between metacognitive self-regulation and cognitive activation (*r* = 0.21, *p* < 0.001).

### 3.2. Preliminary Psychometric Analyses

#### 3.2.1. Assessing Potential Common Method Bias

To ascertain the possibility of common method bias, two analytic approaches were followed, namely exploratory factor analysis and bifactor modelling. The first unrotated factor from an exploratory factor analysis explained 25% of the variance in the indicators, which, according to some guidelines, suggests that common method bias is unlikely to dominate the results (Howard & Henderson, 2023). From the bifactor modelling, the ECV for the general method factor was 0.20 and the omega hierarchical coefficient of reliability for this method factor was ω_H_ = 0.31. The ECV indicates that the method factor captured only 20% of the variance across all items and exhibited low reliability. This is taken to suggest that there is not a dominant general response factor.

#### 3.2.2. Confirmatory Factor Analysis of the Measurement Model

In the first instance, CFAs were implemented to evaluate the construct validity of each measure separately. To evaluate the construct validity, the strength of the factor loadings and the goodness-of-fit indices were inspected. The separate CFAs for each multi-item measure indicated that all measures utilised in the present study exhibited acceptable construct validity. The goodness-of-fit indices are presented in Table 2 below. The latent factor loadings were all above 0.4 and many in the range of 0.5 to 0.8, indicating strong correspondence between the specified items and the hypothesised constructs. The factor loadings and the item wordings can be found in Tables S1–S3 in Supplementary Materials.

Next, all measures were then pooled in a CFA measurement model, which exhibited acceptable fit to the data, namely χ^2^ (130) = 1124.444, *p* < 0.001, CFI = 0.918, TLI = 0.903, RMSEA = 0.035 90%CI [0.033–0.036], SRMR = 0.051. This indicates that the hypothesised underlying measurement structure is sufficient to support structural modelling.

### 3.3. Structural Model of the Process Linking Cognitive Activation to Mathematics Self-Efficacy

**Figure 3:**
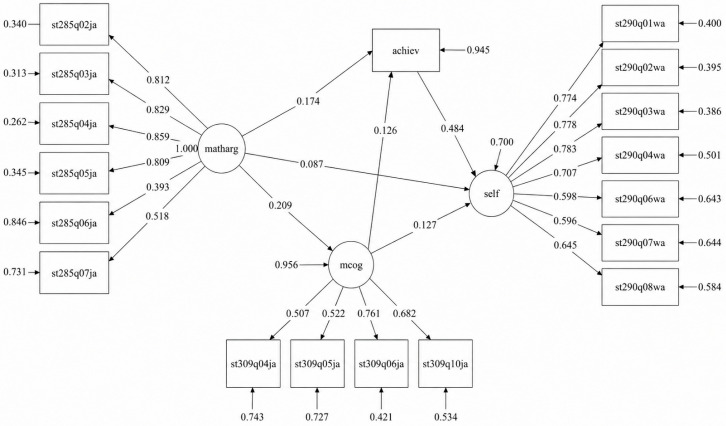
Full path diagram of the structural equation model. Matharg: Cognitive activation—mathematics argumentation (explaining and justifying); MCOG: Metacognitive self-regulation; Self: Mathematics self-efficacy; ACHIEV: PISA 2022 standardised mathematics performance; directed arrows indicate regressions; only statistically significant paths are shown (at least *p* < 0.05).

The model’s fit to the data was acceptable with χ^2^ (130) = 1124.444, *p* < 0.001, CFI = 0.918, TLI = 0.903, RMSEA = 0.035 90%CI [0.033–0.036], SRMR = 0.051. The final SEM model is presented in Figure 3, where standardised regression coefficients that reached statistical significance are presented. As can be seen in Figure 3, the results indicated a coherent pattern of associations from cognitive activation—mathematics argumentation (MATHARG) to metacognitive self-regulation (MCOG) (β = 0.209, *p* < 0.001). In turn, metacognitive self-regulation was positively associated with mathematics performance (β = 0.126, *p* < 0.001) and mathematics self-efficacy (β = 0.126, *p* < 0.001). Cognitive activation—mathematics argumentation also exerted a direct effect on mathematics performance (β = 0.174, *p* < 0.001) and mathematics self-efficacy (β = 0.087, *p* < 0.001). Furthermore, mathematics performance emerged as the strongest factor associated with mathematics self-efficacy (β = 0.484, *p* < 0.001). This pattern is consistent with the mastery-experiences account. Overall, the model explained 30%, 5%, and 4% of the variance in mathematics self-efficacy, performance, and metacognitive self-regulation, respectively, indicating that the model accounted for substantially more variance in self-efficacy than in the other factors.

Indirect effects were computed to examine the mediating pathways within the model. For mathematics performance, the total effect of cognitive activation—mathematics argumentation (MATHARG) was statistically significant (*β* = 0.201, *p* < 0.001), comprising both a direct effect (*β* = 0.174, *p* < 0.001) and a small but significant indirect effect through metacognitive self-regulation (MCOG) (*β* = 0.026, *p* < 0.001). This indicates that approximately 13% of the total effect of cognitive activation on mathematics performance is explained via metacognitive self-regulation. For mathematics self-efficacy, the total effect of MATHARG was also significant (*β* = 0.211, *p* < 0.001), with a sizeable proportion accounted for by indirect pathways (*β* = 0.124, *p* < 0.001). Specifically, three significant indirect effects were identified: (a) via mathematics performance (*β* = 0.084, *p* < 0.001), (b) via metacognitive self-regulation (*β* = 0.027, *p* < 0.001), and (c) a sequential pathway through metacognitive self-regulation and mathematics performance (*β* = 0.013, *p* < 0.001). These findings suggest that approximately 59% of the total effect of cognitive activation on mathematics self-efficacy was accounted for by statistically significant indirect associations. Taken together, the results highlight mathematics performance as the primary mediator linking cognitive activation to self-efficacy, while metacognitive self-regulation was involved in an additional cross-sectional indirect association. Details about the specific indirect pathways are presented in Table 3.

Table 3 shows several notable patterns regarding the associations between cognitive activation, metacognitive self-regulation, mathematics performance and self-efficacy. Regarding mathematics performance, the indirect association between cognitive activation—mathematics argumentation (MATHARG) and performance is modest but statistically significant, operating through metacognitive self-regulation (MCOG) (*β* = 0.026, *p* < 0.001). This finding suggests that cognitively activating instructional practices are positively associated with better regulatory processes, which in turn correlate with higher mathematics performance. However, the majority of the total association remained direct, suggesting that cognitive activation was linked with performance through pathways not fully accounted for by metacognitive self-regulation.

For mathematics self-efficacy, the total indirect effect was *β* = 0.124, *p* < 0.001, accounting for approximately 59% of the total effect. This indicates that the relationship between cognitive activation and self-efficacy is largely indirect, although a significant direct effect remains. The pattern of specific indirect effects reveals multiple meaningful pathways. First, the strongest indirect association operated through mathematics performance (*β* = 0.084, *p* < 0.001), consistent with the role of mastery experiences as a key source of self-efficacy. This finding indicates that cognitively activating instructional practices are associated with higher confidence in students’ mathematical abilities primarily through their association with mathematics performance. Second, a significant indirect association emerged through metacognitive self-regulation (*β* = 0.027, *p* < 0.001), suggesting that students’ capacity to regulate their learning processes also correlates independently with self-efficacy beliefs. Finally, a sequential mediation pathway was supported, whereby cognitive activation was associated with metacognitive self-regulation, which in turn was related to better performance, and subsequently to higher self-efficacy (*β* = 0.013, *p* < 0.001). These findings should be interpreted as evidence of theoretically ordered, cross-sectional associations rather than as evidence of a developmental or causal process. Within this associational model, mathematics performance represented the strongest intervening factor in the association between cognitive activation and self-efficacy, whereas metacognitive self-regulation showed a smaller but statistically significant association both directly and through mathematics performance.

## 4. Discussion

The present study examined the interplay between cognitive-activating instruction, metacognitive self-regulation, mathematics performance and mathematics self-efficacy from a social-cognitive perspective. Drawing on a large, nationally representative dataset from PISA 2022, the study provided several important insights into the underlying psychological processes that are associated with mathematics self-efficacy beliefs, while also clarifying the links between cognitive-activating instruction and metacognitive regulation. In brief, the results of the structural modelling highlight that stimulating and challenging instructional practices were associated with internal regulatory processes which were, in turn, positively associated with mathematics performance and subsequent self-efficacy beliefs. In the following sections, the findings are discussed and linked with previous studies.

### 4.1. Cognitive-Activating Instruction as an Important Correlate of Metacognitive Regulation and Mathematics Performance

The results of the modelling revealed several patterns in the associations of cognitive-activating instruction with metacognitive regulation, and mathematics performance. The link between cognitive-activating instruction and mathematics performance is well established (Li et al., 2021; Zuo et al., 2024); however, evidence that explicitly links cognitive-activating instruction with metacognitive self-regulation remains limited. Cognitive-activating instruction has been linked in the past with deep thinking and challenging learning environments that create opportunities for students’ learning (Ekatushabe et al., 2021; Li et al., 2021). However, only a few studies have provided concrete and direct evidence in favour of a positive association between cognitive-activating instruction (e.g., questioning, modelling, applying previous knowledge) and metacognition (Ekatushabe et al., 2021; Kyriakides et al., 2020).

As was initially hypothesised, cognitive activation in the form of mathematics argumentation was statistically significantly associated with metacognitive self-regulation with a modest effect size. Hence, hypothesis H1 was supported. This finding seems to suggest that students’ ability to reflect on and check their mathematics learning might be a critical metacognitive process that can be boosted by cognitive-activating instruction. Thus, the social-cognitive logic underpinning the study’s model is supported because an instructional strategy, which is an environmental factor, is directly related to internal metacognitive regulatory processes. Overall, the current findings directly underscore the relevance of cognitive-activating instruction for adolescent students’ levels of metacognitive self-regulation in mathematics.

Within the broader model, cognitive activation was associated with mathematics performance both directly and indirectly through metacognitive self-regulation. However, the indirect effect was relatively modest, indicating that while metacognitive self-regulation plays a meaningful role, cognitive activation also exerts a sizeable direct influence on performance.

### 4.2. Metacognitive Self-Regulation and Mathematics Self-Efficacy

Metacognitive self-regulation is widely recognised as a key process that supports effective learning since it enables students to actively plan, monitor, and control their cognitive and behavioural strategies (Oudman et al., 2022; Rivers, 2021). The present findings provide empirical support for this role within the context of mathematics learning. Hence, hypothesis 2 was supported. Within the current social-cognitive framework of self-efficacy, metacognitive self-regulation occupies a dual role. Beyond its well-known role in predicting mathematics performance (Fu & Qi, 2025; Wang et al., 2021), metacognitive self-regulation was directly and statistically significantly associated with mathematics self-efficacy. This finding is theoretically meaningful because it suggests that mathematics self-efficacy beliefs are informed not only by performance-based mastery experiences, but also by adolescents’ capacity to self-evaluate and control their own learning process.

From a social-cognitive perspective, this direct association may represent the role of internal self-evaluative processes in belief formation. Metacognitive self-regulation encompasses processes such as monitoring, strategy adjustment, and self-evaluation, which together provide learners with internal feedback about their learning progress (Efklides, 2008). Students who actively regulate their learning by planning, adjusting strategies and persisting in mathematics may develop a stronger sense of personal agency and control over their mathematics learning (Schunk & DiBenedetto, 2020; Zimmerman et al., 2017). This, in turn, can foster more positive mathematics self-efficacy beliefs, even beyond the influence of objective performance outcomes (Bandura, 1991; Usher & Pajares, 2009). Importantly, this finding aligns well with a separate strand of research that indicates that students rely on internal judgments about their performance (e.g., confidence judgments, perceived understanding) when forming beliefs about their competence (Dunlosky & Metcalfe, 2009; Rivers, 2021). Whilst mathematics performance provides objective external evidence of competence that can increase mathematics self-efficacy beliefs, metacognitive self-regulation skills may provide internal feedback about the mathematics learning progress, which students may use when forming judgments about their competence (Schunk & DiBenedetto, 2020).

The findings suggest that metacognitive self-regulation may be associated with self-efficacy through students’ internal self-evaluative processes, although the cross-sectional design precludes definitive conclusions about whether metacognitive regulation functions as an independent source of self-efficacy. In the next section, I discuss the final part of the model, which connects performance to self-efficacy.

### 4.3. Performance as a Potential Source of Self-Efficacy

The final and key outcome of the current study is mathematics self-efficacy. Mathematics self-efficacy has long been conceived as an important antecedent of mathematics performance (Street et al., 2024). Yet, social-cognitive theory indicates that mathematics performance can serve as mastery experiences, which increase students’ mathematics self-efficacy beliefs (Usher, 2009; Usher & Pajares, 2009). In fact, mastery experiences have been shown to be the strongest predictor of high mathematics self-efficacy (Peura et al., 2026), a finding which clearly aligns with the current study’s model. Hence, hypothesis 3 was supported.

Despite the cross-sectional nature of the data, the current result regarding the association between mathematics performance and self-efficacy contributes tentatively to the wider discussion on the links between these two important factors. Specifically, it supports the broader social-cognitive perspective (Bandura, 1991; Schunk & DiBenedetto, 2020), by providing concrete process evidence consistent with a pathway from an external environmental influence (cognitive activation) to internal personal regulatory processes (metacognitive self-regulation) to a behavioural outcome (mathematics performance), which may contribute to the formation of personal self-beliefs (mathematics self-efficacy). Moreover, previous empirical research has questioned the extent to which performance can predict self-efficacy (Schöber et al., 2018) and other studies point toward a small association (Du et al., 2021; R. Liu et al., 2024). Yet, the current study, despite its design limitations due to the cross-sectional nature of the data, provided evidence in favour of a moderate effect size.

Overall, the findings emphasise the need to conceptualise mathematics self-efficacy as a construct that can be informed by mastery experiences in the classroom context. By placing mathematics self-efficacy at the end of the social-cognitive process linking cognitive-activating instruction, metacognitive self-regulation, and mathematics performance, the study provides a more holistic account of the factors associated with self-efficacy beliefs. In fact, the model suggests that mathematics self-efficacy beliefs do not form in isolation from the wider cognitively and instructionally driven processes. Based on the findings, I turn next to the educational implications and the future directions for research.

### 4.4. Limitations and Future Directions for Research

The present study has several limitations. First, it is recognised that the PISA 2022 data, despite being nationally representative and valid, remain cross-sectional and cannot support causal explanations. Thus, the discussion of the findings should be interpreted in light of this limitation. Second, the metacognitive self-regulation measure was operationalised and formally validated within the current study’s design. Thus, this measure requires further refinement in future studies, particularly given that metacognitive self-regulation was assessed using a brief proxy measure and showed comparatively modest CFA fit. Third, the relationship between mathematics performance and self-efficacy is probably best represented as bidirectional. Hence, future research should examine the directionality of this relationship via longitudinal designs or feedback effects. Fourth, it was necessary to modify the original PISA 2022 cognitive activation scale due to construct validity concerns in this Greek sample. This indicates that the modified measure might not be entirely comparable to the original scale, but it remains valid for national use within Greece. Finally, although the model explained a significant proportion of the variance in self-efficacy, there is unexplained variance in mathematics performance and metacognitive self-regulation, suggesting that other factors need to be considered in future research. Additional student-, classroom- and school-level factors not included in the present model are likely to play an important role. Moreover, since metacognitive self-regulation was operationalised through a brief proxy measure, the construct may not have been captured as comprehensively as in studies using dedicated metacognitive measures.

### 4.5. Implications for Practice

In terms of educational implications, the study offers several cautious considerations for educational practice, especially in light of the consistent declines in academic performance among Greek adolescents over the years (I. Katsantonis et al., 2023). First, the findings suggest that instructional environments that prioritise cognitive activation may be associated with metacognitive self-regulation, mathematics performance and self-efficacy. Second, although the current study does not involve an intervention design, it shows that metacognitive self-regulation may be one factor associated with both mathematics performance and self-efficacy. Thus, emphasising metacognitive self-regulation strategies may be useful for helping students evaluate their thinking processes and mathematical work and detect errors, although further intervention research is needed to determine whether such strategies improve mathematics performance or self-efficacy beliefs. Third, given the positive association between mathematics performance and self-efficacy, it may be important to design instructional environments that provide opportunities for students to experience and reflect on successful mathematics performance, which is consistent with the role of mastery experiences in self-efficacy development (Özcan & Kültür, 2021). Finally, the findings indicate that it might be useful to reconsider the role of metacognitive self-regulation in adolescence, particularly within the Greek context. Rather than implying that metacognitive self-regulation can directly alleviate declines in academic performance, the present findings suggest that it may be a relevant factor to consider in future longitudinal and intervention research on mathematics performance and self-efficacy.

## 5. Conclusions

In conclusion, the study provided a theoretically grounded test of associations among cognitively activating instruction, adolescents’ mathematics self-efficacy, metacognitive self-regulation, and mathematics performance. The key findings highlight mathematics performance as the strongest behavioural correlate within the proposed social-cognitive model. Additionally, the study suggests that metacognitive self-regulation may be a modest complementary correlate of mathematics self-efficacy within the broader pattern of associations among cognitive-activating instruction, mathematics performance, and self-efficacy. By situating mathematics self-efficacy within a broader social-cognitive framework, the current study contributes to the literature by providing nationally representative correlational evidence from Greek PISA 2022 data. Overall, the results are consistent with the view that instructional environments that involve deep thinking and reasoning, together with metacognitive self-regulatory processes, are meaningfully associated with mathematics performance and self-efficacy.

## Figures and Tables

**Table 1:** Descriptive statistics for the key variables under study.

Variable	Mean	SD	Minimum	Maximum	Skewness	Reliability Coefficient
Mathematics Self-efficacy	2.48	0.76	1	4	0.01	0.87
Metacognitive Self-regulation	3.65	0.86	1	5	−0.62	0.72
Cognitive Activation: Mathematics Argumentation	3.16	1.07	1	5	−0.19	0.85
Mathematics Performance	430.14	79.23	128.1	702.44	0.24	0.89

**Table 2:** Goodness-of-fit indices of the CFA models per measure.

Measure	Scaled Chi-Square (df)	CFI	TLI	RMSEA	SRMR
Cognitive activation: Mathematics argumentation	124.685 (9) ***	0.968	0.946	0.047	0.049
Metacognitive self-regulation	49.758 (2) ***	0.925	0.774	0.063	0.053
Mathematics self-efficacy	150.415 (14) ***	0.967	0.950	0.041	0.039

Note: *** *p* < 0.001; CFI: Comparative fit index; TLI: Tucker–Lewis index; RMSEA: Root mean square error of approximation; SRMR: Standardised root mean residual.

**Table 3:** Standardised specific indirect paths in the SEM model for the key outcomes.

Path	Coefficient (S.E.)	*p*-Value
**Effects on Performance**		
Argumentation → Metacognitive Self-regulation → Performance	0.026 (0.006)	0.000
**Effects on Self-efficacy**		
Argumentation → Performance → Self-efficacy	0.084 (0.011)	0.000
Argumentation → Metacognitive Self-regulation → Self-efficacy	0.027 (0.006)	0.000
Argumentation → Metacognitive Self-regulation → Performance → Self-efficacy	0.013 (0.003)	0.000
**Direct Effect**		
Argumentation → Self-efficacy	0.087 (0.018)	0.000

Note: Outcome variables are in bold.

## Data Availability

Data are available from the Organization for Economic Co-operation and Development at https://www.oecd.org/en/data/datasets/pisa-2022-database.html (accessed on 23 July 2024).

## Supplementary Materials

The following supporting information can be downloaded at: https://www.mdpi.com/article/10.3390/bs16061029/s1, Table S1: Factor Loadings of the Self-regulation Scale; Table S2: Factor Loadings of the Cognitive Activation Scale: Mathematics Argumentation; Table S3: Factor Loadings of the Mathematics Self-efficacy Scale.
