# Supplementary material for: Relating Cognitive-Activating Instruction and Metacognitive Self-Regulation to Mathematics Performance and Self-Efficacy: A Process-Modelling Study
Source: Behav Sci (Basel). 2026 Jun 19;16(6):1029. doi: 10.3390/bs16061029 (PMC13295810; doi:10.3390/bs16061029)
Supplement: Supplementary file 1 [file behavsci-16-01029-s001.zip › behavsci-4322896-supplementary.pdf]

Supplemental Materials

Table S1. Factor Loadings of the Self-regulation Scale

	**Metacognitive Self-regulation** (Strategy use, regulation of learning)		
**Code**	**Item wording**	**Reverse?**	**Factor Loading (coefficient)**
ST309Q06JA	I stop to think before acting.	No	.775***
ST309Q05JA	I carefully check homework before turning it in.	No	.499***
ST309Q10JA	I think carefully before doing something.	No	.722***
ST309Q04JA	I like to make sure there are no mistakes	No	.462***

*** *p*<.001

Table S2. Factor Loadings of the Cognitive Activation Scale: Mathematics Argumentation

**Item Code**	**Item Wording**	**Factor Loading (coefficient)**
ST285Q02JA	The teacher asked us to explain how we solved a mathematics problem.	.806***
ST285Q03JA	The teacher asked us to explain what assumptions we were making when solving a mathematics problem.	.834***
ST285Q04JA	The teacher asked us to explain our reasoning when solving a mathematics problem.	.861***
ST285Q05JA	The teacher asked us to defend our answer to a mathematics problem.	.804***
ST285Q06JA	The teacher asked us to think about how new and old mathematics topics were related.	.401***
ST285Q07JA	The teacher encouraged us to think about how to solve mathematics problems in different ways than demonstrated in class.	.523***

The initial PISA 2022 scale included 9 items (ST285Q01JA- ST285Q09JA). Based on Confirmatory Factor Analysis (CFA) of these items for the Greek sample, item ST285Q01JA had a standardised factor loading of .103 and was dropped. The CFA fit after dropping the 1st item was RMSEA= .059, SRMR= .071, CFI= .901, TLI= .869. Thus, the modification indices were inspected. The modification indices suggested residual correlations and endogeneity between items 8 and 9. Thus, items ST285Q08JA and ST285Q09JA were dropped and the fit

indices reached very good levels of fit: RMSEA= .047, SRMR= .049, CFI= .968, TLI= .946. This explanation has been added in the supplemental materials.

Table S3. Factor Loadings of the Mathematics Self-efficacy Scale

Item Code	Item Wording	Factor Loadings (coefficient)
**ST290Q01WA**	Calculate the petrol consumption rate of a car.	.753***
**ST290Q02WA**	Calculate how much cheaper a TV would be after a 30% discount.	.759***
**ST290Q03WA**	Calculate how many square metres of tiles you need to cover a floor.	.772***
**ST290Q04WA**	Understand graphs presented in newspapers.	.733***
**ST290Q06WA**	Solve an equation like 3x + 5 = 17.	.635***
**ST290Q07WA**	Calculate the average speed of a vehicle.	.548***
**ST290Q08WA**	Solve a word problem involving proportions or percentages.	.682***
